# Oxytocin-Induced Acute Pulmonary Edema: A Case Report and Literature Review

**DOI:** 10.7759/cureus.15067

**Published:** 2021-05-16

**Authors:** Mohamed K Mansour, Mohamed Dehelia, Yousif M Hydoub, Omar Kousa, Babar Hassan

**Affiliations:** 1 Internal Medicine, Sheikh Shakhbout Medical City, Abu Dhabi, ARE; 2 Cardiology, Sheikh Khalifa Medical City, Abu Dhabi, ARE; 3 Internal Medicine, Creighton University, Omaha, USA; 4 Pulmonology, Sheikh Shakhbout Medical City, Abu Dhabi, ARE

**Keywords:** postpartum dyspnea, oxytocin, acute pulmonary edema

## Abstract

The use of intravenous (IV) oxytocin has been commonly associated with the development of nausea, vomiting, headache, flushing, and hypotension. To date, only a few previously published studies have linked the administration of IV oxytocin, in high doses exceeding 15 mU/min, with the development of acute pulmonary edema. In this article, we aim to report the rare occurrence of acute pulmonary edema following administration of IV oxytocin at a small dose of 2 mU/min, in a 20-year-old pregnant female, to allow its recognition and prompt treatment by the clinician caring for the patient.

## Introduction

Oxytocin is an oligopeptide hormone that is synthesized in the hypothalamus; however, it is stored in and released from the posterior pituitary gland. It is one of the few hormones that work by the positive feedback mechanism. Its functions include stimulation of uterine contractions as well as stimulation of the myoepithelial cells in the female breast causing milk expression [1]. In clinical practice, exogenous oxytocin is approved for use in the antepartum period to strengthen uterine contractions, to facilitate vaginal delivery of the fetus. In the partum period, it approved for use in the third stage of labor to allow delivery of the placenta and prevent postpartum hemorrhage [2]. Common adverse effects associated with the administration of oxytocin include flushing, nausea, vomiting, and headache, in addition to hypotension, from peripheral vasodilation, as well as compensatory tachycardia [3]. Oxytocin has also been shown to have antidiuretic properties, owing to its similar chemical structure to the antidiuretic hormone (vasopressin), and a few studies, in fact, have associated administration of oxytocin in high doses (above 15 mU/min) with water intoxication and hyponatremia [4-7]. Furthermore, our literature review has revealed that there are only four case reports of oxytocin-induced acute pulmonary edema [8-11]. In this article, we report a case of acute pulmonary edema developing in a 20-year-old pregnant female, following the use of intravenous (IV) oxytocin for induction of labor, thereby adding to the limited available literature on the subject.

## Case presentation

**Figure 1:**
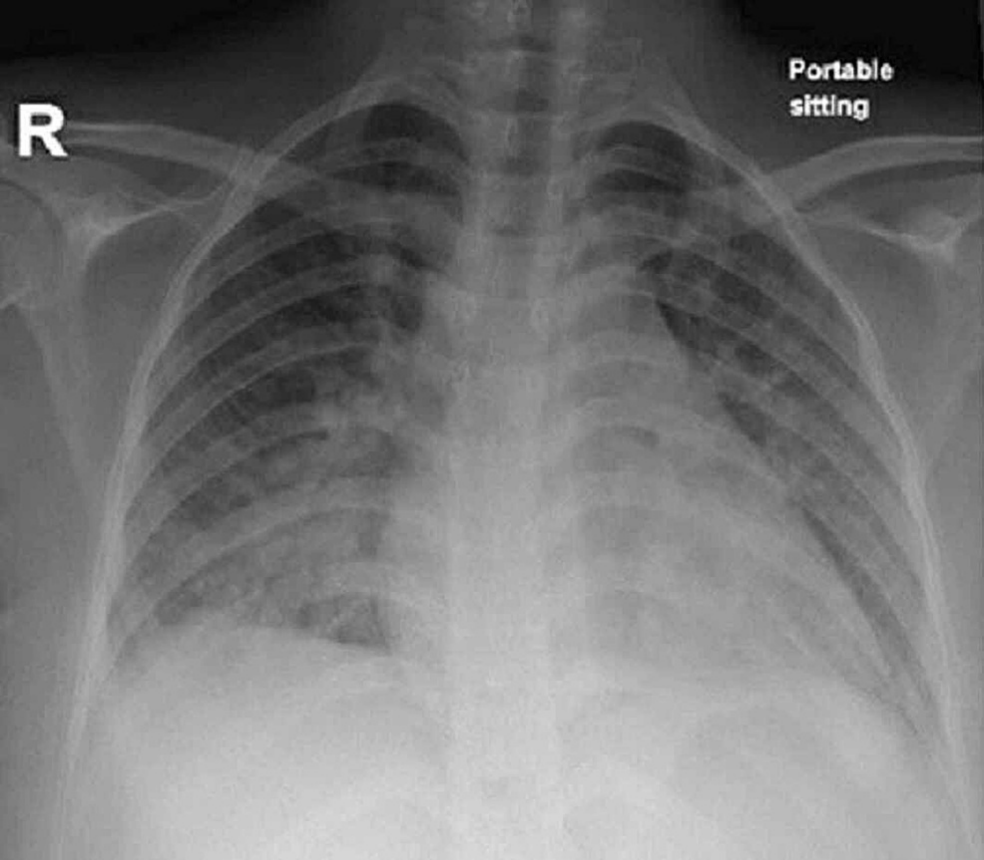
Portable anteroposterior chest X-ray showing evidence of pulmonary edema following administration of oxytocin.

The patient was a 20-year-old female, 29 weeks pregnant, who was admitted to the obstetrics and gynecology service of our hospital with preterm premature rupture of membranes. She was previously healthy and had no history of chronic medical conditions. Three days after admission, a trial of induction of labor was attempted by administration of 10 units of oxytocin in 500 ml lactated ringer’s solution at a rate of 2 mU/min for five hours. This resulted in successful vaginal delivery of the fetus. Four hours after delivery, the patient suddenly started to complain of shortness of breath and chest discomfort. She had no fever, cough, or sputum production. She has a respiratory rate of 30 breaths per minute. Oxygen saturation was 95% on room air. Blood pressure was normal. There was no evidence of jugular venous distention. Chest auscultation revealed the presence of bilateral inspiratory crepitations. There was no swelling or erythema of the lower limbs. Laboratory tests showed a serum sodium level of 139 mEq/L (136-145), potassium level of 3.6 mEq/L (3.5 to 5.1), chloride level of 106 mEq/L (96 to 106), bicarbonate level of 23 mEq/L (23 to 30), creatinine of 0.38 mg/dL (0.50-0.90), WBC of 14,580 cells/dL (4,000-11,000), hemoglobin of 10 g/dL (11 to 13), C‐reactive protein (CRP) of 33.8 mg/L (<5), N-terminal pro-brain natriuretic peptide (NT-pro-BNP) of 1234 pg/mL (0-130), troponin T of 5.52 ng/L (<14), D-dimer of 5.77 microgram/mL (<0.5). A chest X-ray done on the patient revealed evidence of pulmonary edema as shown in Figure 1.

**Figure 2:**
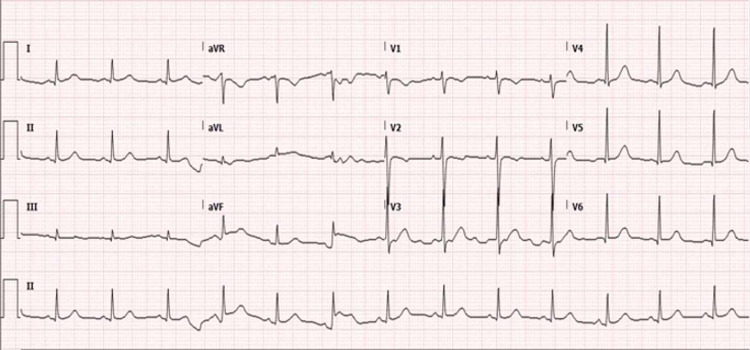
Electrocardiogram showing sinus rhythm and no acute ischemic changes.

Total fluid intake over the last 24 hours was 2.3 liters. Urine output, over the same period of time, was 1.8 liters. Polymerase chain reaction (PCR) test for severe acute respiratory syndrome coronavirus-2 (SARS-CoV-2) on a nasopharyngeal swab was negative. An electrocardiogram showed normal sinus rhythm with no ischemic changes or evidence of right heart strain as demonstrated in Figure 2.

**Figure 3:**
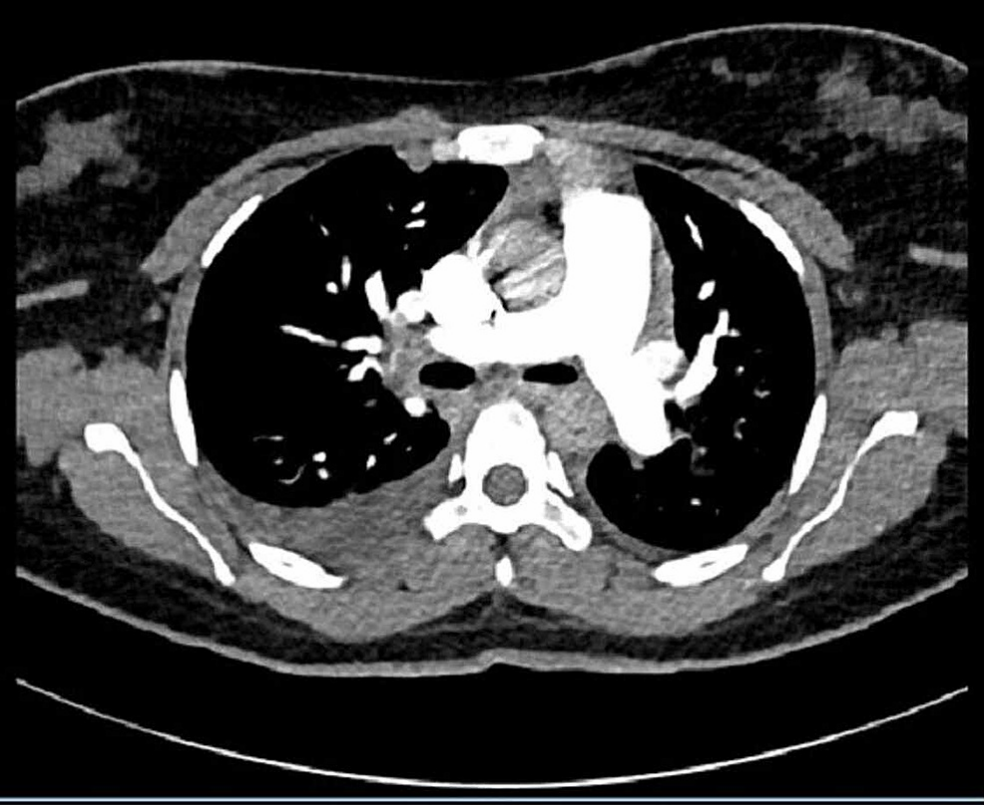
CT pulmonary angiogram showing no evidence of pulmonary embolism.

CT pulmonary angiogram was done as shown in Figure 3. It was reported as showing a small right-sided pleural effusion, no consolidation, and no evidence of pulmonary embolism.

**Figure 4:**
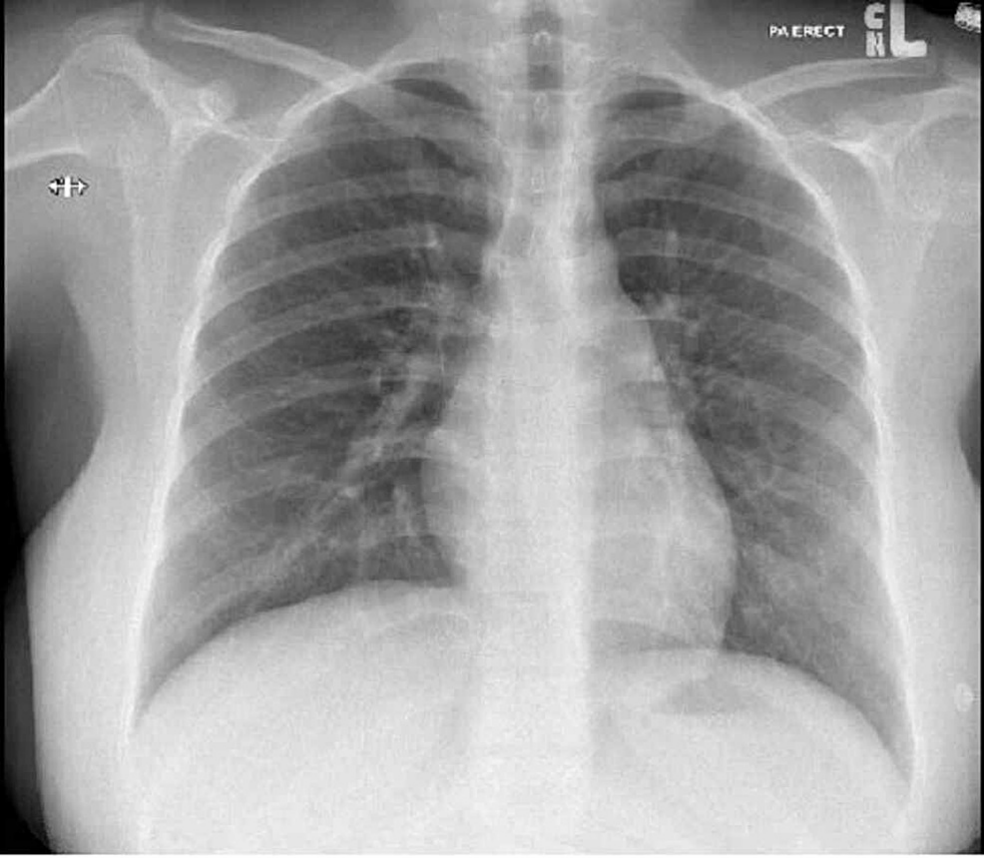
Erect posteroanterior chest X-ray showing resolution of pulmonary edema after diuresis with intravenous furosemide.

Transthoracic echocardiography showed normal left ventricular systolic function with an ejection fraction of 60%. Furthermore, it showed no evidence of valvular heart disease or pericardial effusion. The patient was given 20 mg of furosemide intravenously every 12 hours for one day. She reported an improvement in her symptoms after the first dose of IV furosemide. High vaginal swab culture obtained on the day of admission grew normal vaginal flora. A repeat chest X-ray done three days later showed complete resolution of the pulmonary edema as seen in Figure 4, and hence the patient was discharged home in good health. The patient was scheduled to have a follow-up appointment at the clinic after discharge, but unfortunately, she did not attend the scheduled appointment.

## Discussion

Several studies in the literature have described the antidiuretic properties of oxytocin by reporting cases of pregnant patients developing water intoxication, particularly manifesting as acute dilutional hyponatremia, following oxytocin administration [4-6]. However, to date, only a few reports have described cases of pregnant females developing acute pulmonary edema following oxytocin administration. Heytens (1984) and Shahin (1991) described cases of pregnant females who had developed acute pulmonary edema after administration of IV oxytocin, used to control postpartum hemorrhage [8-9]. Ghai et al. (2006) reported a case of a 26-year-old primigravida who had developed acute severe pulmonary edema following delivery, after use of IV oxytocin for induction of labor [10]. A case report by Dogdu (2010) described a 34-year-old, 17 weeks pregnant, female patient who was initially admitted with acute lateral myocardial infarction. The patient was started on IV oxytocin infusion for termination of pregnancy due to intrauterine fetal death. Following that, she developed acute pulmonary edema. The patient was given a total of 80 mg of IV furosemide, however, she remained in respiratory distress, which necessitated intubation and mechanical ventilation. The patient developed cardiac arrest 24 hours later, and despite aggressive resuscitative measures, she passed away [11].

Oxytocin effects on the cardiovascular system have been extensively studied. The presence of oxytocin receptors has been demonstrated in the heart and the vasculature. Oxytocin has been shown to cause a decrease in blood pressure via a vasodilatory effect for a short period of time, which results in transient cardiac ischemia. Oxytocin also exerts a transient negative chronotropic and ionotropic effect on the heart [12]. The combination of decreased heart rate, decreased cardiac contractility, and decreased cardiac perfusion, coupled with the antidiuretic properties of the hormone, can be postulated as a possible mechanism underlying the genesis of pulmonary edema in patients receiving IV oxytocin [7,12].

In this case, the development of acute pulmonary edema was attributed to the use of IV oxytocin infusion for induction of labor. Our patient had no evidence of pneumonia or pulmonary embolism on chest imaging. A cardiac etiology for her symptoms was ruled out with the help of an electrocardiogram, which showed no cardiac arrhythmias and no evidence of cardiac ischemia. Moreover, echocardiography demonstrated a normal cardiac function and no valvular lesions, thereby eliminating peripartum cardiomyopathy and valvular heart disease as possible causes for her symptoms, respectively. This leaves oxytocin-induced pulmonary edema as a plausible explanation for her symptoms.

This case is unique in that it adds to the limited available literature describing oxytocin-induced pulmonary edema. Furthermore, this condition had previously been described with the administration of IV oxytocin infusion in high doses exceeding 15 mU/min. This case goes to show that the condition can develop in parturient females even with the administration of small doses of IV oxytocin (2 mU/min), using non-electrolyte based free fluids, as vehicles for the administration of the drug.

## Conclusions

Administration of IV oxytocin can be a possible cause for the development of acute pulmonary edema in the parturient female, particularly in the absence of other more common causes of this condition. The antidiuretic effect of oxytocin may occur, even with the administration of small doses of the drug. Further studies with a stronger evidence base are needed to prove the association between oxytocin administration and the development of acute pulmonary edema.

## References

[1] Bhargava R, Daughters KL, Rees A (2019). Oxytocin therapy in hypopituitarism: challenges and opportunities. Clin Endocrinol.

[2] Simpson KR (2011). Clinicians' guide to the use of oxytocin for labor induction and augmentation. J Midwifery Womens Health.

[3] Dyer RA, Butwick AJ, Carvalho B (2011). Oxytocin for labour and caesarean delivery: implications for the anaesthesiologist. Curr Opin Anaesthesiol.

[4] Pittman JG (1963). Water intoxication due to oxytocin-report of a case. N Engl J Med.

[5] Liggins GC (1962). The treatment of missed abortion by high dosage syntocinon intravenous infusion. J Obstet Gynaecol Br Emp.

[6] Ahmad AJ, Clark EH, Jacobs HS (1975). Water intoxication associated with oxytocin infusion. Postgrad Med J.

[7] Li C, Wang W, Summer SN, Westfall TD, Brooks DP, Falk S, Schrier RW (2008). Molecular mechanisms of antidiuretic effect of oxytocin. J Am Soc Nephrol.

[8] Heytens L, Camu F (1984). Pulmonary edema during cesarean section related to the use of oxytocic drugs. Acta Anaesthesiol Belg.

[9] Shahin J, Guharoy SR (1991). Pulmonary edema possibly developing secondary to the intravenous administration of oxytocin. Vet Hum Toxicol.

[10] Ghai B, Vayjnath AM, Lal S (2006). Acute pulmonary oedema following oxytocin administration: a life threatening complication. J Indian Med Assoc.

[11] Dogdu O, Yarlioglues M, Inanc T, Ardic I, Zencir C, Kaya MG (2011). Fatal pulmonary oedema following oxytocin administration in a pregnant woman with acute myocardial infarction. Cardiovasc Toxicol.

[12] Gutkowska J, Jankowski M, Antunes-Rodrigues J (2014). The role of oxytocin in cardiovascular regulation. Braz J Med Biol Res.

